# Comparative Analysis of Calcium Spikes upon Activation of Serotonin_1A_ and Purinergic Receptors

**DOI:** 10.1371/journal.pone.0051857

**Published:** 2012-12-19

**Authors:** Roopali Saxena, Sourav Ganguly, Amitabha Chattopadhyay

**Affiliations:** Centre for Cellular and Molecular Biology, Council of Scientific and Industrial Research, Hyderabad, India; Medical School of Hannover, United States of America

## Abstract

Calcium signaling represents one of the most important signaling cascades in cells and regulates diverse processes such as exocytosis, muscle contraction and relaxation, gene expression and cell growth. G protein-coupled receptors (GPCRs) are the most important family of receptors that activate calcium signaling. Since calcium signaling regulates a large number of physiological responses, it is intriguing that how changes in cytosolic calcium levels by a wide range of stimuli lead to signal-specific physiological responses in the cellular interior. In order to address this issue, we have analyzed temporal calcium profiles induced by two GPCRs, the serotonin_1A_ and purinergic receptors. In this work, we have described a set of parameters for the analysis of calcium transients that could provide novel insight into mechanisms responsible for maintaining signal specificity by shaping calcium transients. An interesting feature of calcium signaling that has emerged from our analysis is that the profile of individual transients in a calcium response could play an important role in maintaining downstream signal specificity. In summary, our analysis offers a novel approach to identify differences in calcium response patterns induced by various stimuli.

## Introduction

Calcium signaling represents one of the most important signaling cascades in cells. The indispensability of calcium signaling is apparent from the fact that it governs a large number of processes in cells [1]–[3]. In the cytosol, calcium is maintained at a very low level (∼100 nM) and is concentrated in intracellular calcium stores such as the endoplasmic reticulum (ER) and extracellular spaces (∼2 mM) [3]. The difference in calcium concentration between the cytosol and these intracellular stores or extracellular spaces could be utilized very effectively to deliver signals across the cell membrane. This implies that any stimulus that activates calcium signaling leads to release of calcium from these stores and a subsequent increase in cytosolic calcium concentration [4]. Extracellular stimuli that initiate calcium signaling could either activate voltage-gated calcium channels present in the plasma membrane or induce the activation of ligand-gated calcium channels present on the intracellular stores of calcium [5], [6].

The G protein-coupled receptor (GPCR) superfamily is the largest and most diverse protein family in mammals, involved in signal transduction across membranes [7], [8]. Since GPCRs regulate multiple physiological processes, they have emerged as major targets for the development of novel drug candidates in all clinical areas [9]. It is estimated that ∼50% of clinically prescribed drugs act as either agonists or antagonists of GPCRs [10]. GPCRs represent the most important family of receptors that activate calcium signaling by activating ligand-gated calcium channels present in the intracellular stores of calcium. The general mechanism through which GPCRs initiate calcium signaling involves the activation of phospholipase C (PLC) through activated heterotrimeric G proteins upon ligand-mediated stimulation [11]. PLC catalyzes the conversion of phosphatidylinositol 4,5-bisphosphate (PIP_2_) present in the plasma membrane into inositol 1,4,5-triphosphate (IP_3_) and diacylglycerol (DAG). Subsequently, IP_3_ diffuses into the cytoplasm and binds to calcium channels termed as IP_3_ receptors present on ER membranes, thereby releasing calcium into the cytoplasm [6]. The released calcium in the cytosol is either actively taken up by ER through calcium channels called sarco/endoplasmic reticulum Ca^2+^-ATPase (SERCA), or effluxed out of the cell through calcium channels present on the plasma membrane. These processes lead to the interesting occurrence of calcium spikes/transients which involves repetitive rise and decay of calcium concentration in the cytosol. During a brief increase of calcium in the cytoplasm, it initiates a series of signaling events through activation of various proteins such as calmodulin [12].

As mentioned above, calcium signaling regulates diverse processes such as exocytosis, muscle contraction and relaxation, gene expression and cell growth. Given its involvement in such a diverse set of processes, it is intriguing how changes in cytosolic calcium levels lead to signal-specific physiological responses inside cells. Although literature related to calcium signaling has tremendously increased in the past two decades, understanding the signal specificity of calcium signaling continues to be the most challenging problem. For example, calcium has been shown to regulate both relaxation and contraction of vascular smooth muscle depending on its spatial distribution inside smooth muscle [13]. It has been suggested that specific spatial and temporal distribution of intracellular calcium upon receptor activation lead to such specific physiological responses [14]. The presence of stimulus (such as ligand) presents two major challenges to cells, *i.e.*, identification of its nature and strength. A simple strategy could be activation and modulation of pathways that are sensitive to the characteristics of stimulus. In this overall context, it is extremely difficult to experimentally elucidate and assign mechanisms involved in the identification of various signals, due to the inherent complexity and cross-talk of signaling pathways encountered in cells [15]. In addition, since the process of calcium release and uptake upon signaling occurs over a timescale of a few hundreds of milliseconds, it often makes it difficult to visualize and analyze the elementary events of the process. Simulation studies therefore have become increasingly popular in order to understand the underlying mechanism involved in shaping of ligand-induced calcium response [16]. Although simulation studies have provided novel insight into the mechanisms of calcium responses, their validation in biological system has proven to be challenging. In order to explore this issue, we have utilized fast fluorescence imaging to acquire calcium transients and developed an analysis approach for the temporal calcium profiles induced by two GPCRs, serotonin_1A_ and purinergic receptors.

The serotonin_1A_ receptor is an important neurotransmitter receptor and couples to inhibitory G protein (Gα_i_) [17]. Serotonergic signaling is implicated in the generation and modulation of various cognitive, behavioral and developmental functions [18]. On the other hand, purinergic receptors respond to adenosine nucleotides and couple to Gα_q_ in CHO cells [19]. Activation of purinergic receptors results in multitude of physiological effects which could range from neurotransmission to inflammatory responses [20]. Both receptors utilize calcium cascade to bring about their specific physiological responses. In this work, we have analyzed calcium spikes induced by ligand-mediated stimulation of the serotonin_1A_ and purinergic receptors (P_2_ receptors) in Chinese Hamster Ovary (CHO) cells stably expressing the human serotonin_1A_ receptor (termed as CHO-5-HT_1A_R). While serotonin_1A_ receptors are stably transfected, the purinergic receptor is endogenously present in these cells. We have identified parameters of calcium transients that are sensitive to the ligand concentration. In addition, our analysis highlights the differences in the characteristics of calcium spikes induced by different ligands. Our results constitute one of the first reports on the characterization of individual transient of calcium in order to decipher the ligand-specific signature of calcium response. These results would provide useful insights into processes involved in shaping the calcium profile in response to different ligands/stimuli.

## Materials and Methods

### Materials

Serotonin, ATP, penicillin, streptomycin, gentamycin sulfate, and pluronic acid were obtained from Sigma Chemical Co. (St. Louis, MO). DMEM/F-12 (Dulbecco's modified Eagle's medium), fetal calf serum, geneticin (G 418) and fluo-3/AM were from Invitrogen Life Technologies (Carlsbad, CA). The μ-Slides I^0.4^ Luer were from Ibidi (Martinsried, Germany). All other chemicals used were of the highest purity available. Water was purified through a Millipore (Bedford, MA) Milli-Q system and used throughout.

### Cells and cell culture

CHO cells stably expressing the serotonin_1A_ receptor (termed CHO-5-HT_1A_R cells) were maintained in D-MEM/F-12 (1∶1) supplemented with 2.4 g/l of sodium bicarbonate, 10% fetal calf serum, 60 µg/ml penicillin, 50 µg/ml streptomycin, 50 µg/ml gentamycin sulfate, and 200 µg/ml geneticin in a humidified atmosphere with 5% CO_2_ at 37°C.

### Loading of CHO-5-HT_1A_R cells with fluo-3/AM

Cells (∼5×10^4^) were plated in μ-slides and grown for 24 h. Loading of cells with fluo-3/AM was carried out by incubating cells in serum-free D-MEM/F-12 (1∶1) medium containing 10 µM fluo-3/AM and 0.02% (v/v) pluronic acid for 15 min at room temperature (∼25°C). After incubation, cells were washed twice with PBS and calcium signaling was monitored upon ligand addition. Changes in cytosolic calcium level were visualized immediately after loading cells with fluo-3/AM in cases where ATP was used as a ligand. Serotonin-induced calcium response was imaged after incubation of fluo-3/AM loaded cells for 45 min at room temperature (∼25°C).

### Imaging of ligand-induced calcium signaling in CHO-5-HT_1A_R cells

Imaging of calcium response was carried out on an inverted Zeiss LSM 510 Meta confocal microscope (Jena, Germany) at room temperature (∼25°C). Fluo-3/AM loaded cells were imaged in PBS with 20×/0.75 NA objective using the 488 nm line of an argon laser and fluorescence of calcium-bound fluo-3 was collected using 500–550 nm filter. Images were recorded every 0.25 sec for a total time of ∼3.3 min with bidirectional scanning without averaging to achieve maximum temporal resolution. Ligand was added on the cells in μ-slides on stage after the acquisition of ∼10 frames.

### Data analysis

The analysis and plotting of data were performed using Origin software version 7.0 (OriginLab Corp., Northampton, MA) and Microsoft Excel 2007. In order to explore the effect of ligand concentration on calcium signaling, we monitored calcium response with a range of ligand concentration. The half maximal effective concentration (EC_50_) was determined upon fitting the data with a range of concentrations of ligand using the four parameter logistic equation as follows:
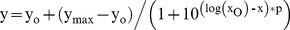
(1)where y is the calcium response in terms of the number of calcium spikes observed per cell, y_o_ is the basal response, y_max_ is the maximal response, x_o_ is the EC_50_ value for ligand, x is the ligand concentration, and p is the slope factor. Further analysis of calcium transients was divided into two categories (see below).

**Figure 1:**
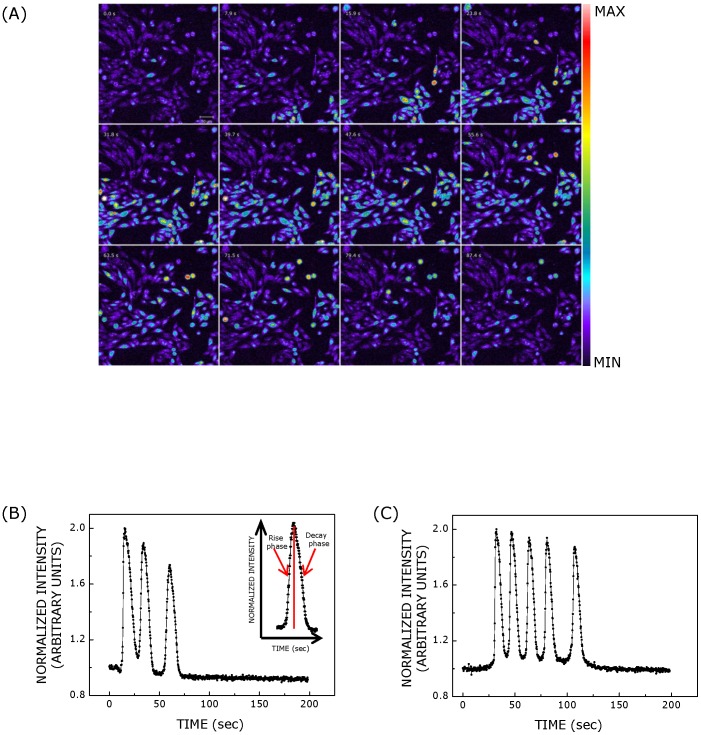
Calcium response in CHO-5-HT_1A_R cells upon stimulation with serotonin or ATP. Panel (A) shows successive images of calcium response in cells upon stimulation with serotonin acquired with a time interval of 7.9 sec. CHO-5-HT_1A_R cells were loaded with Fluo-3/AM to monitor changes in cytosolic calcium concentration. Time-lapse imaging was performed with a 20×/0.75 NA objective. The scale bar (shown in top left panel) represents 50 µm. Similar response was observed when cells were stimulated with ATP. Representative temporal profiles of calcium response induced by either (B) serotonin or (C) ATP are shown. The concentration of serotonin used was 100 µM and that of ATP was 500 nM, respectively. Both serotonin and ATP induce the occurrence of multiple spikes consisting of rise and decay phases. A typical calcium spike with rise and decay phases is shown in the inset in panel (B). The temporal profile of calcium response was intensity normalized such that the maximum and minimum (basal) intensities corresponded to values 2 and 1, respectively. See Materials and Methods for more details.

The first category consists of parameters that are described for the dependence of calcium response on ligand concentration. These parameters include maximum-fold increase in the fluorescence of fluo-3/AM upon ligand-mediated increase in cytosolic calcium level (F_max_/F_o_) and the apparent time constant (τ) for the release of calcium in the cytoplasm from intracellular stores. F_o_ and F_max_ represent fluorescence intensities of calcium-bound fluo-3/AM corresponding to basal and ligand-induced maximum cytosolic calcium concentrations, respectively. Since increase in the fluorescence of fluo-3/AM is directly proportional to the amount of calcium present, F_max_/F_o_ would correspond to the maximum-fold increase in calcium concentration upon receptor activation. In order to explore the kinetics of release and uptake of calcium in the cytoplasm, we divided calcium transient into two phases, *i.e.*, rise and decay phases (see inset in Fig. 1B). We chose to analyze the kinetics of only rise phase of the first spike of calcium response (which consists of multiple spikes) since we often observed overlapping spikes, particularly at higher ligand concentrations. The time constant of the rise phase for the first calcium transient was obtained after fitting the normalized data (such that F_o_ = 1 and F_max_ = 2) to a single exponential model as:
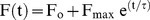
(2)where F(t) is the fluorescence intensity of calcium-bound fluo-3/AM at time t, F_o_ is the basal fluorescence intensity of calcium-bound fluo-3/AM, F_max_ is the maximum fluorescence intensity of calcium-bound fluo-3/AM upon ligand addition and τ is the apparent time constant for the increase in the fluorescence intensity of calcium bound fluo-3/AM. We evaluated peak characteristics by using the simplest model for spike functions, i.e., Gaussian function (equation 3). It should be noted that these models do not incorporate the molecular mechanism of calcium signaling and give the average parameter for phenomena happening inside cells.

The second category consists of parameters that are described for the dependence of calcium response on ligand type. These parameters include amplitude, full width at half maxima (FWHM), and area of calcium transients. The temporal profile of calcium transients was normalized (such that F_o_ = 1 and F_max_ = 2) prior to obtaining these parameters. After normalization, the temporal profile of calcium was fitted with a multi-Gaussian function considering each spike to be Gaussian in nature. The function used to fit the spikes in the temporal profile of calcium response is:
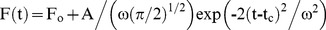
(3)where F(t) is the fluorescence intensity of calcium-bound fluo-3/AM at time t, F_o_ is the background fluorescence intensity of calcium-bound fluo-3/AM, A is the area under the spike, and t_c_ is the time point corresponding to the maximum fluorescence intensity of calcium-bound fluo-3/AM after ligand addition and ω is full width of the spike at half maximal response (FWHM). It should be noted here that the assumption about Gaussian shape of calcium transient is an approximation. In reality, the shape of calcium transients deviate from a perfect Gaussian profile as the rise phase always appear steeper than the decay phase. In terms of calcium response, the amplitude signifies the maximum-fold increase in cytosolic calcium during each spike. On the other hand, the area corresponds to the total amount of calcium released during each transient and FWHM is proportional to the life span of calcium transient in the cytoplasm. We did not observe any dependence of these parameters on ligand concentration used for respective spikes after normalization. We therefore collated and plotted data obtained for the entire range of ligand concentrations used corresponding to respective calcium spikes.

Eqn. (1) is a well established and commonly used relationship to analyze receptor pharmacology at multiple steps upon ligand binding. Calcium signaling represents one such step and is a downstream process relative to ligand binding to the receptor. We therefore employed eqn. 1 to analyze dose response curves for calcium signaling upon activation of receptors. In order to analyze kinetics of the rise phase and characteristics of calcium spikes, we decided to employ simple models. We therefore chose to evaluate kinetics by employing the equation for monoexponential increase (eqn. (2)). In addition, rise and decay phases of calcium spikes have been recently analyzed in a similar manner in astrocytes and dendritic spines [21], [22]. We evaluated spike characteristics by using the simplest model for spike functions, i.e., Gaussian function (eqn. (3)).

## Results

### Ligand specificity of calcium response: number of calcium transients depends on ligand concentration

**Figure 2:**
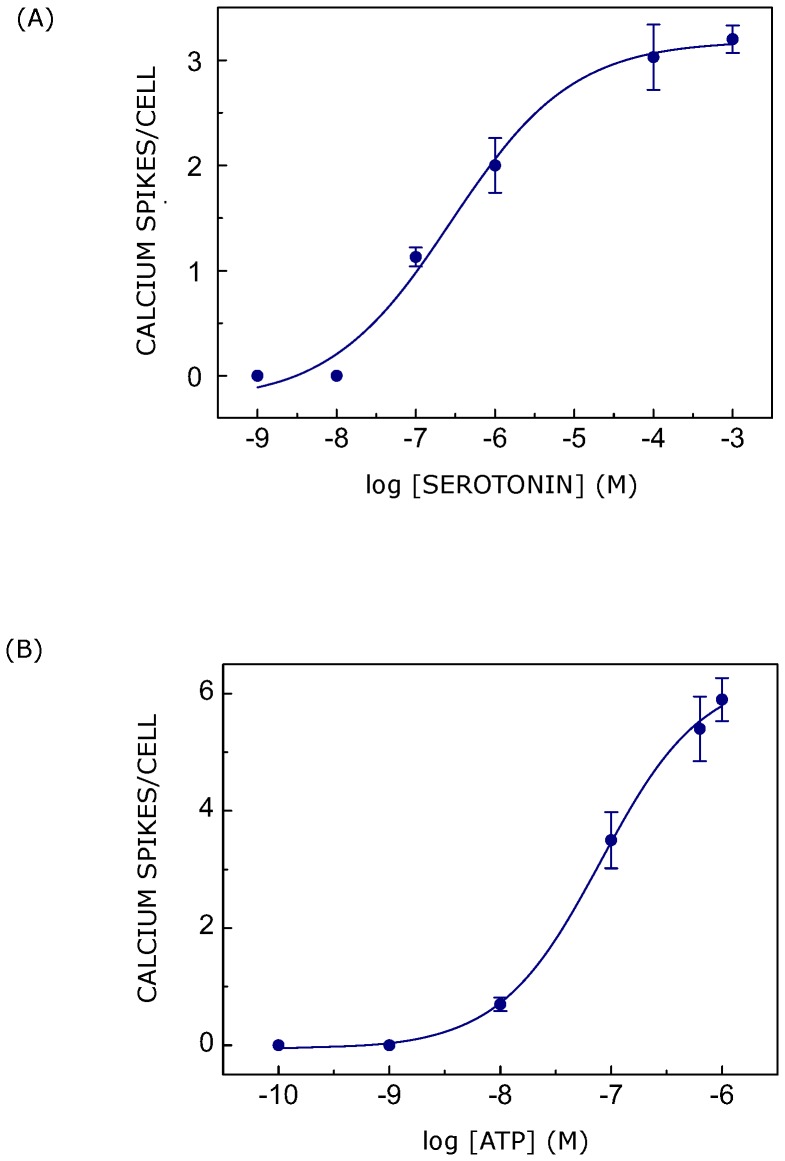
Dose response plots for calcium signaling induced by ligands in CHO-5-HT_1A_R cells. The figure shows calcium response in terms of a number of calcium spikes visualized per cell induced with either (A) serotonin or (B) ATP. The curves are nonlinear regression fits to the experimental data using eqn. 1. Data represent means ± SEM of more than 30 cells from at least four independent experiments. See Materials and Methods for more details.

In order to monitor changes in cytosolic calcium level, we employed a calcium- sensitive fluorophore fluo-3/AM. Fluo-3/AM is a cell permeant variant of a calcium indicator whose fluorescence intensity increases upon binding to calcium [23]. Upon stimulation of fluo-3/AM loaded CHO cells with serotonin or ATP, we observed repetitive calcium transients as opposed to the single broad spike reported earlier [24], [25]. This is possibly due to the fact that we employed higher speed imaging of cells with a temporal resolution of ∼250 ms in contrast to temporal resolution of few sec achieved in earlier reports. The high temporal resolution in our measurements provided us the advantage to visualize ligand-mediated repetitive calcium spikes. Fig. 1A shows a representative series of images scanned after every 7.9 sec showing calcium response in CHO-5-HT_1A_R cells upon stimulation with serotonin. The temporal intensity profile of calcium response in each cell was obtained by analyzing sequence of images acquired after every ∼250 ms. Such temporal profiles of calcium, induced by serotonin and ATP in single cells are shown in Fig. 1B and C. As mentioned earlier, each calcium spike consists of rise and decay phases as shown in the inset of Fig. 1B. The number of calcium spikes visualized per cell showed a dependence on ligand concentration in both cases (see Fig. 2). The figure shows that the number of calcium spikes increased with an increase in ligand concentration. The dose response curves for serotonin and ATP are shown in Fig. 2A and B, respectively. Dose response curves were fitted with eqn. 1 and EC_50_ values were calculated for serotonin and ATP. EC_50_ values obtained were ∼0.28 and ∼0.08 µM in cases of stimulation with serotonin and ATP, respectively. Interestingly, these ligands induced different responses, in terms of number of calcium spikes visualized per cell, corresponding to the same ligand concentration. ATP appeared to be more potent ligand than serotonin in eliciting calcium response. It should be noted that all cells loaded with fluo-3/AM do not exhibit calcium response upon addition of ligand. Their response depends on ligand concentration and type. In addition, the occurrence of oscillations, rather than a single transient, also depends on ligand concentration. For example, probability of occurrence of oscillations increases with increase in ligand concentration.

### Maximum fold change in cytosolic calcium level depends on ligand concentration

**Figure 3:**
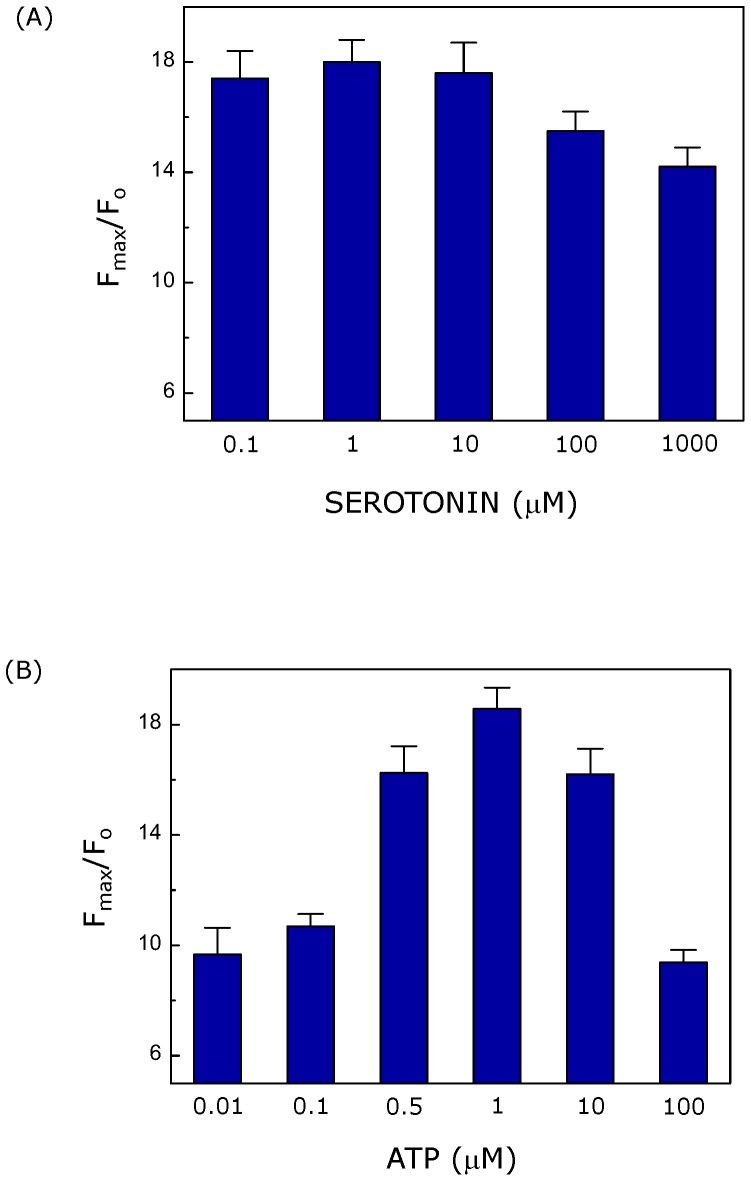
The maximum fold change in cytosolic calcium levels depends on ligand concentration. The figure shows calcium response in terms of maximum fold change in cytosolic calcium levels induced with either (A) serotonin or (B) ATP. Only the first spike of calcium response (when composed of multiple spikes) induced by ligands was analyzed. Data represent means ± SEM of more than 30 cells from at least four independent experiments. See Materials and Methods for more details.

We monitored the maximum fold change in cytosolic calcium concentration (F_max_/F_o_) induced by either ligands (serotonin or ATP) with increasing concentrations. F_max_/F_o_ *vs.* ligand concentration plots corresponding to the first spike (see later for analysis of subsequent spikes) of ligand-induced calcium response are shown in Fig. 3. The fold change in fluorescence (reflecting change in cytosolic calcium) appears more or less independent of ligand concentration in case of serotonin stimulation up to a certain concentration. The maximum response appeared to decrease at higher concentrations of serotonin (see Fig. 3A). It has been earlier reported that higher concentration of calcium inactivates IP_3_ receptors [26]. It is therefore possible that IP_3_ receptors could be deactivated due to high local concentration of calcium, as a result of fast kinetics of calcium release (see later) at higher serotonin concentrations. In contrast, ATP-induced maximum fold change in cytosolic calcium appears to show a bell-shaped profile with increasing ligand concentration. Interestingly, both serotonin and ATP induced maximum calcium response of comparable magnitude (F_max_/F_o_ = ∼18) although at different ligand concentrations.

### Rate of calcium release from intracellular stores depends on ligand concentration

**Figure 4:**
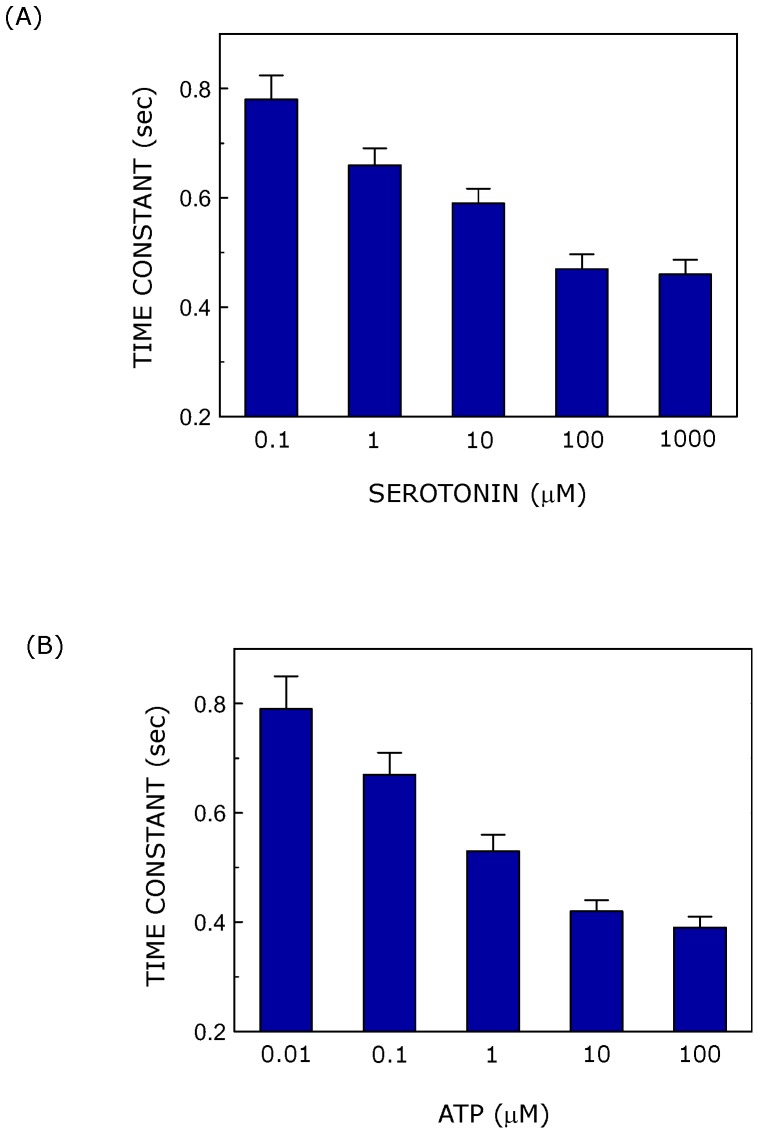
The rate of increase in cytosolic calcium levels depends on ligand concentration. The figure shows the apparent time constant for the rise phase of the first calcium spike induced with varying concentrations of either (A) serotonin or (B) ATP. R^2^ values for fitting of the rise phase of calcium spikes with monoexponential function range from 0.94–1 in all cases. Data represent means ± SEM of more than 30 cells from at least four independent experiments. See Materials and Methods for more details.

We monitored the kinetics of calcium release from intracellular stores with increasing concentrations of either serotonin or ATP. Ligand-induced release of calcium from intracellular stores contributes to the rise phase of calcium spike. The rise phase of each spike was fitted with first order exponential function and the corresponding time constant (τ) was derived. Time constants as a function of ligand concentration, corresponding to the first spike of calcium response, are shown in Fig. 4. Time constants for the rise phase exhibited a reduction with increasing ligand concentration in both cases. For example, time constants obtained were ∼0.78 and ∼0.47 sec, corresponding to serotonin concentration of 0.1 and 100 µM, respectively (Fig. 4A). The time constants were calculated to be ∼0.79 and ∼0.42 sec, corresponding to 0.01 and 10 µM ATP (Fig. 4B). Interestingly, the time constants appear to plateau at higher ligand concentrations. This limiting value of time constant (∼0.4 sec) could correspond to the fastest rate of calcium response.

### Dependence of amplitude, FWHM and area of calcium spike on its position

**Figure 5:**
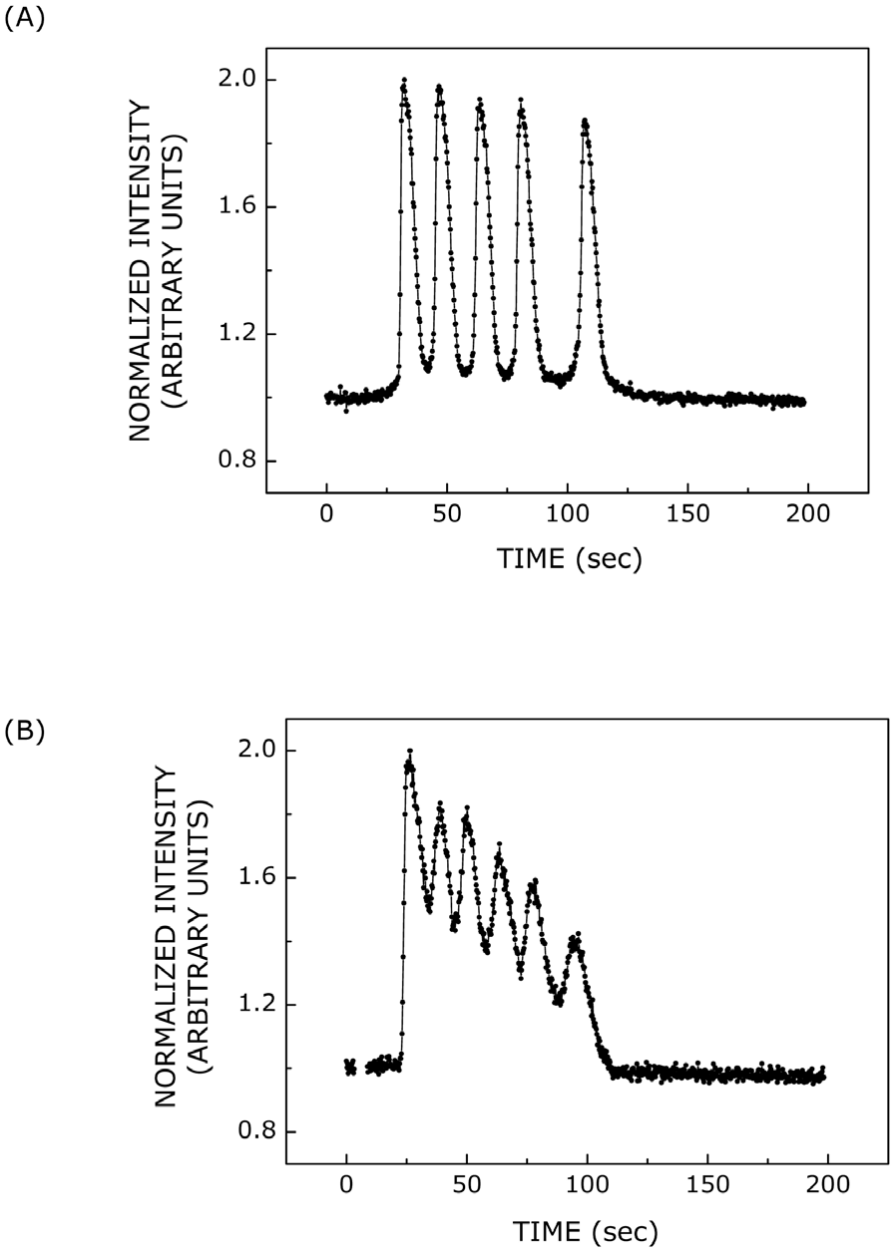
Calcium responses induced by ATP. Representative (A) distinct or (B) overlapping calcium spikes observed upon stimulation with 500 nM ATP. The propensity of the occurrence of overlapping calcium spikes increased with increase in ATP concentration. The temporal profile of calcium response was intensity-normalized such that the maximum and minimum (basal) intensities corresponded to values 2 and 1, respectively. See Materials and Methods for more details.

**Figure 6:**
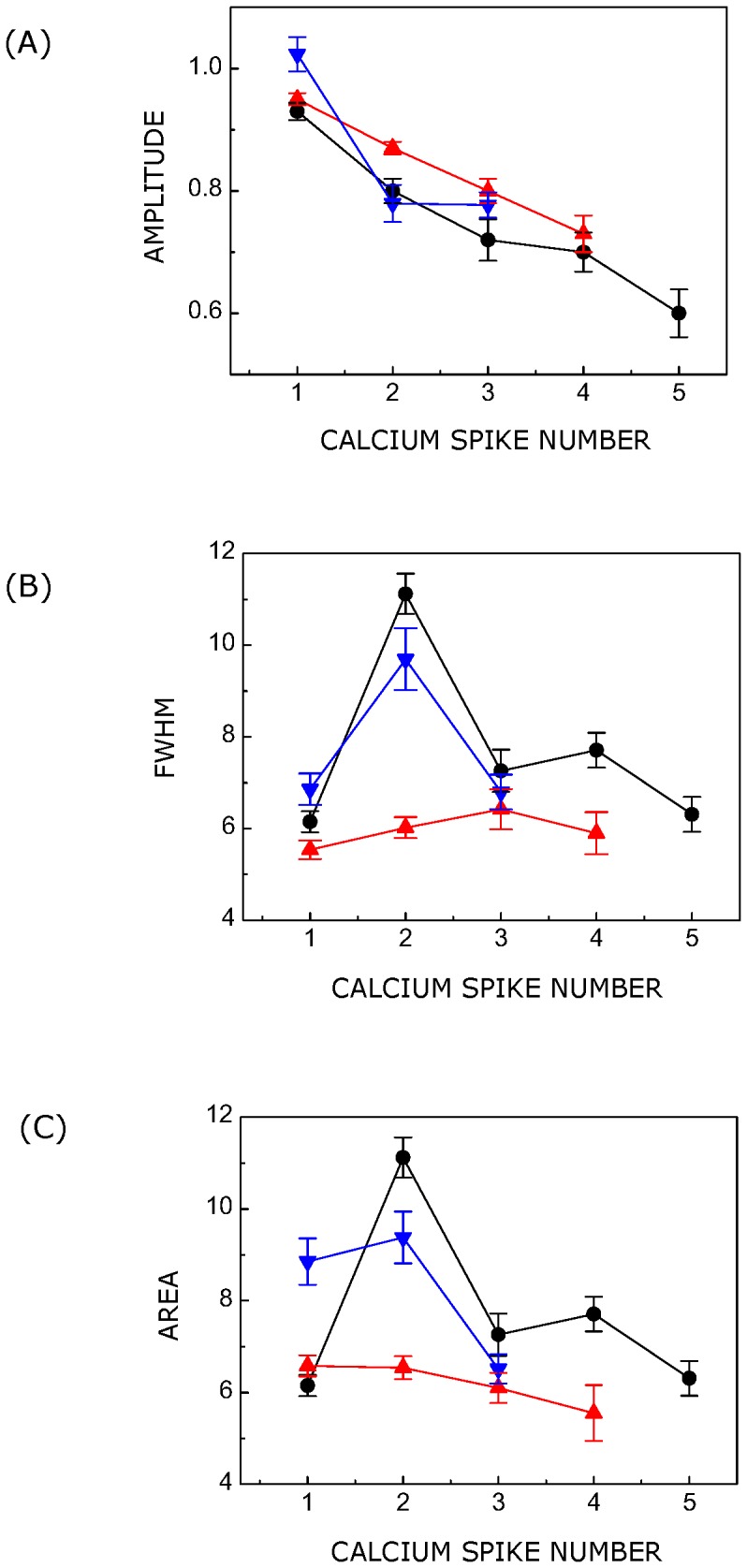
Amplitude, full width at half maxima (FWHM) and area vary for individual spike in a multi-spike response. The amplitude of calcium spike represents the maximum fold change in cytosolic calcium level. The area of calcium spike corresponds to the amount of calcium released in the cytoplasm and FWHM denotes the life span of the calcium spike in the cytoplasm. Panels (A), (B) and (C) show amplitude, FWHM and area for serotonin-induced calcium spikes (blue triangle), and ATP-induced distinct (red triangle) and overlapping (•) calcium spikes, respectively. The temporal profile of calcium response was intensity-normalized such that the maximum and minimum (basal) intensities corresponded to values 2 and 1, respectively, before fitting it with a multi-gaussian function. In case of serotonin and ATP-stimulated calcium spikes, R^2^ values for fitting of calcium spikes with Gaussian function ranged from 0.92–0.98 and 0.94–0.99, respectively. Values represent means ± SEM of more than 50 cells from at least four independent experiments. See Materials and Methods for more details.

We observed two different (distinct or overlapping) types of calcium response induced by serotonin or ATP. In the case of distinct spikes, each spike is clearly separated from its adjacent spikes. On the other hand, overlapping spikes are characterized by partial overlap of a spike with its adjacent spikes. In general, serotonin induced distinct calcium spikes. Calcium response induced by ATP, however, displayed both types of spikes (see Fig. 5). The probability of overlapping spikes increased with increase in ligand concentration. We monitored the dependence of amplitude, full width at half maxima (FWHM) and area of calcium spike on spike position. In case of serotonin stimulation, the amplitude (maximum fold change in cytosolic calcium) showed a reduction in case of second spike and remained similar for third spike. On the other hand, ATP-induced distinct and overlapping spikes displayed almost linear reduction in the amplitude of subsequent spikes (see Fig. 6A). FWHM signifies the lifespan of ligand-induced increase in cytosolic calcium concentration. FWHM is governed by all processes involved in rise and decay phases of calcium spikes. Fig. 6B shows the dependence of FWHM on spike position in ligand-induced calcium response. Both serotonin-induced spikes and ATP-mediated overlapping spikes showed an increase in FWHM for the second spike. On the other hand, FWHM of ATP-mediated distinct spikes was found to be more or less invariant with respect to spike position. The area under the spike represents the total amount of calcium released during the event of calcium spike. In addition, the area is dependent on both amplitude and FWHM of the spike. Fig. 6C shows the dependence of the area of calcium spike on its position in ligand-induced calcium response. The amount of calcium released was found to be similar for first two spikes and decreased for the third spike in case of serotonin-mediated response. In case of ATP-mediated overlapping spikes, the amount of calcium released was highest for the second spike. On the other hand, the area of ATP-mediated distinct spikes was found to be less sensitive to spike position.

## Discussion

In the present work, we have analyzed serotonin- and ATP-induced periodic changes in cytosolic calcium levels (temporal calcium profiles) in CHO cells. We have described a set of parameters for calcium transients that could provide novel insight into mechanisms responsible for maintaining the specificity of signals by shaping calcium transients. Our results suggest that the maximum fold change in cytosolic calcium concentration and the kinetics of calcium release from intracellular calcium stores into the cytoplasm depend on ligand concentration. In addition, we demonstrated that the amplitude, duration and area of spike vary with its number (position) in the temporal profile of calcium. Importantly, we observed variation in the amplitude, duration and area of the respective spikes induced by different ligands.

GPCRs mediate multiple physiological processes such as neurotransmission, cellular metabolism, secretion, cellular growth and differentiation, and immune response [27]. However, only a limited number of second messengers, which brings about specific outcomes in response to stimulation of various receptors present on cell membranes, have been identified [28]. This has given rise to the challenge of understanding the coupling of various stimuli to their signaling counterparts, which produces specific physiological responses. In this context, calcium signaling represents a universal and important constituent of such signaling networks. For example, it has been shown that acetylcholine and glucose mobilize calcium from different intracellular calcium stores by agonist-specific coupling of second messengers in MIN6 cells [29]. Since GPCRs regulate multiple physiological processes, they have emerged as major targets for the development of novel drug candidates in all clinical areas [9]. As mentioned earlier, it is estimated that ∼50% of clinically prescribed drugs act as either agonists or antagonists of GPCRs [10]. Interestingly, although GPCRs represent ∼50% of current drug targets, only a small fraction of all GPCRs are presently targeted by drugs [30]. This raises the exciting possibility that the receptors that are not recognized yet could be potential drug targets for diseases that are difficult to treat by currently available drugs. In this overall context of GPCRs in cellular signaling and as drug targets, studies aimed at understanding the underlying mechanisms involved in shaping of receptor-induced calcium responses assume relevance.

The universal mode of calcium signaling in cells is characterized by periodic changes in cytosolic calcium levels termed as oscillations [31]. Oscillations confer the much needed specificity on calcium signal, otherwise highly diverse and universal in nature. This signal-specific information is therefore encoded in the spatiotemporal signature of calcium response. For example, the frequency of calcium oscillations controls the expression of many genes such as IL2 and IL8 by differentially regulating the activity of various transcription factors such as NF-AT, Oct/OAP and NF-**κ**B [32], [33]. In response to various stimuli, changes in cytosolic calcium are brought about by coordinated opening and closing of a variety of calcium channels. The shape of a calcium spike therefore would largely be governed by the probability and cooperativity of channel opening. Another important determinant of the shape of calcium spike is subcellular and cell-type dependent spatial distribution and expression level of calcium channels [34]. Further, it has been shown that calcium signaling cross-talks with cAMP signaling which is regulated by spatial organization of cAMP domains [35]. In addition, calcium-binding proteins present in the cytoplasm play an important role in determining calcium response [4]. These proteins are spatially distributed in the cytoplasm and have a wide range of binding affinities for calcium and control the diffusion of calcium in the cytoplasm [36]. Moreover, receptors utilize different signaling counterparts in their downstream signaling pathways. For example, different GPCRs can couple to different G-proteins and interact with diverse effectors such as GRKs. Taken together, all these processes could be important in maintaining the signal specificity of calcium response.

Parameters described by us in the present study such as amplitude, FWHM and area of the calcium spike could account for shape determinants of calcium spikes. Our analysis of ATP- and serotonin-induced temporal profile of calcium suggests that these ligands induce different patterns of calcium signaling. Another interesting feature of calcium signaling that has emerged from our analysis is that the profile of individual transients in a calcium response could play an important role in maintaining downstream signal specificity. This is because our analysis showed that parameters such as amplitude, FWHM and area of the calcium spike varied with spike position for different ligands. Taken together, our analysis provides a novel approach to identify differences in patterns of calcium responses induced by various stimuli. The novelty of our approach lies in analysis of individual spikes in a multi-spike pattern. Existing approaches mainly consider parameters such as frequency of oscillations and overall pattern of calcium response to describe differences between calcium responses induced by various receptors or ligands. To the best of our knowledge, this is one of the first reports which suggest that the shape of individual spikes could vary in a multi-spike calcium response and this could represent an important determinant for the specificity of calcium signaling. In addition, our analysis could be useful in understanding the mechanism of interplay of various determinants of calcium signaling, which confers signal specificity to calcium response.

## References

[1] Carafoli E (2002) Calcium signaling: A tale for all seasons. Proc Natl Acad Sci USA 99: 1115–1122. 11830654 10.1073/pnas.032427999 PMC122154

[2] Berridge MJ, Bootman MD, Lipp P (1998) Calcium – a life and death signal. Nature 395: 645–648. 9790183 10.1038/27094

[3] Clapham DE (2007) Calcium signaling. Cell 131: 1047–1058. 18083096 10.1016/j.cell.2007.11.028

[4] Berridge MJ, Lipp P, Bootman MD (2000) The versatility and universality of calcium signalling. Nat Rev Mol Cell Biol 1: 11–21. 11413485 10.1038/35036035

[5] Hosey MM, Lazdunski M (1988) Calcium channels: molecular pharmacology, structure and regulation. J Membr Biol 104: 81–105. 2903935 10.1007/BF01870922

[6] Berridge MJ, Irvine RF (1989) Inositol phosphates and cell signalling. Nature 341: 197–205. 2550825 10.1038/341197a0

[7] Unal H, Karnik SS (2012) Domain coupling in GPCRs: the engine for induced conformational changes. Trends Pharmacol Sci 33: 79–88. 22037017 10.1016/j.tips.2011.09.007 PMC3273637

[8] Rosenbaum DM, Rasmussen SGF, Kobilka BK (2009) The structure and function of G-protein-coupled receptors. Nature 459: 356–363. 19458711 10.1038/nature08144 PMC3967846

[9] Heilker R, Wolff M, Tautermann CS, Bieler M (2009) G-protein-coupled receptor-focused drug discovery using a target class platform approach. Drug Discov Today 14: 231–240. 19121411 10.1016/j.drudis.2008.11.011

[10] Schlyer S, Horuk R (2006) I want a new drug: G-protein-coupled receptors in drug development. Drug Discov Today 11: 481–493. 16713899 10.1016/j.drudis.2006.04.008

[11] Kiselyov K, Shin DM, Muallem S (2003) Signalling specificity in GPCR-dependent Ca^2+^ signalling. Cell Signal 15: 243–253. 12531423 10.1016/s0898-6568(02)00074-8

[12] Carafoli E (2005) Calcium – a universal carrier of biological signals. FEBS J 272: 1073–1089. 15720383 10.1111/j.1742-4658.2005.04546.x

[13] Nelso'n MT, Cheng H, Rubart M, Santana LF, Bonev AD, et al (1995) Relaxation of arterial smooth muscle by calcium sparks. Science 270: 633–637. 7570021 10.1126/science.270.5236.633

[14] Dupont G, Combettes L, Leybaert L (2007) Calcium dynamics: spatio-temporal organization from the subcellular to the organ level. Intl Rev Cytol 261: 193–245. 10.1016/S0074-7696(07)61005-5 17560283

[15] Bhalla US, Iyengar R (1999) Emergent properties of networks of biological signaling pathway. Science 283: 381–387. 9888852 10.1126/science.283.5400.381

[16] Schuster S, Marhl M, Höfer T (2002) Modelling of simple and complex calcium oscillations: from single-cell responses to intercellular signaling. Eur J Biochem 269: 1333–1355. 11874447 10.1046/j.0014-2956.2001.02720.x

[17] Pucadyil TJ, Kalipatnapu S, Chattopadhyay A (2005) The serotonin_1A_ receptor: a representative member of the serotonin receptor family. Cell Mol Neurobiol 25: 553–580. 16075379 10.1007/s10571-005-3969-3 PMC11529526

[18] Müller CP, Carey RJ, Huston JP, De Souza Silva MA (2007) Serotonin and psychostimulant addiction: focus on 5-HT_1A_ receptors. Prog Neurobiol 81: 133–178. 17316955 10.1016/j.pneurobio.2007.01.001

[19] Marcet B, Chappe V, Delmas P, Verrier B (2004) Pharmacological and signaling properties of endogenous P2Y1 receptors in cystic fibrosis transmembrane conductance regulator-expressing Chinese hamster ovary cells. J Pharmacol Exp Ther 309: 533–539. 14742736 10.1124/jpet.103.063396

[20] Ralevic V, Burnstock G (1998) Receptors for purines and pyrimidines. Pharmacol Rev 50: 413–492. 9755289

[21] Cornelisse LN, van Elburg RAJ, Meredith RM, Yuste R, Mansvelder HD (2007) High speed two-photon imaging of calcium dynamics in dendritic spines: consequences for spine calcium kinetics and buffer capacity. PLoS ONE 2: e1073. 17957255 10.1371/journal.pone.0001073 PMC2034355

[22] James LR, Andrews S, Walker S, de Sousa PRS, Ray A, et al (2011) High-throughput analysis of calcium signalling kinetics in astrocytes stimulated with different neurotransmitters. PLoS ONE 6: e26889. 22046396 10.1371/journal.pone.0026889 PMC3201978

[23] Minta A, Kao JPY, Tsien RY (1989) Fluorescent indicators for cytosolic calcium based on rhodamine and fluorescein chromophores. J Biol Chem 264: 8171–8178. 2498308

[24] Iradale PA, Hill SJ (1993) Increases in intracellular calcium via activation of an endogenous P_2_-purinoceptor in cultured CHO-K1 cells. Br J Pharmacol 110: 1305–1310. 8306069 10.1111/j.1476-5381.1993.tb13960.x PMC2175888

[25] Raymond JR, Albers FJ, Middleton JP (1992) Functional expression of human 5-HT_1A_ receptors and differential coupling to second messengers in CHO cells. Naunyn Schmiedebergs Arch Pharmacol 346: 127–137. 1448178 10.1007/BF00165293

[26] Dawson AP (1997) Calcium signalling: how do IP_3_ receptors work? Curr Biol 7: R544–R547. 9285705 10.1016/s0960-9822(06)00277-6

[27] Pierce KL, Premont RT, Lefkowitz RJ (2002) Seven-transmembrane receptors. Nat Rev Mol Cell Biol 3: 639–650. 12209124 10.1038/nrm908

[28] Parekh AB (2011) Decoding cytosolic Ca^2+^ oscillations. Trends Biochem Sci 36: 78–87. 20810284 10.1016/j.tibs.2010.07.013

[29] Yamasaki M, Masgrau R, Morgan AJ, Churchill GC, Patel S, et al (2004) Organelle selection determines agonist-specific Ca^2+^ signals in pancreatic acinar and β cells. J Biol Chem 279: 7234–7240. 14660554 10.1074/jbc.M311088200

[30] Lin SH, Civelli O (2004) Orphan G protein-coupled receptors: targets for new therapeutic interventions. Ann Med 36: 204–214. 15181976 10.1080/07853890310024668

[31] Millard PJ, Gross D, Webb WW, Fewtrell C (1988) Imaging asynchronous changes in intracellular Ca^2+^ in individual stimulated tumor mast cells. Proc. Natl. Acad. Sci. USA 85: 1854–1858. 3162312 10.1073/pnas.85.6.1854 PMC279879

[32] Dolmetsch RE, Lewis RS, Goodnow CC, Healy JI (1997) Differential activation of transcription factors induced by Ca^2+^ response amplitude and duration. Nature 386: 855–858. 9126747 10.1038/386855a0

[33] Dolmetsch RE, Xu K, Lewis RS (1998) Calcium oscillations increase the efficiency and specificity of gene expression. Nature 392: 933–936. 9582075 10.1038/31960

[34] Vermassen E, Parys JB, Mauger J-P (2004) Subcellular distribution of the inositol 1,4,5-trisphosphate receptors: functional relevance and molecular determinants. Biol Cell 96: 3–17. 15093123 10.1016/j.biolcel.2003.11.004

[35] Bruce JIE, Straub SV, Yule DI (2003) Crosstalk between cAMP and Ca^2+^ signaling in non-excitable cells. Cell Calcium 34: 431–444. 14572802 10.1016/s0143-4160(03)00150-7

[36] Dargan SL, Parker I (2003) Buffer kinetics shape the spatiotemporal patterns of IP_3_-evoked Ca^2+^ signals. J Physiol 553: 775–788. 14555715 10.1113/jphysiol.2003.054247 PMC2343635

